# Identification of three immunodominant motifs with atypical isotype profile scattered over the *Onchocerca volvulus* proteome

**DOI:** 10.1371/journal.pntd.0005330

**Published:** 2017-01-26

**Authors:** Ole Lagatie, Bieke Van Dorst, Lieven J. Stuyver

**Affiliations:** Janssen Diagnostics, Janssen Pharmaceutica NV, Beerse, Belgium; University of Liverpool, UNITED KINGDOM

## Abstract

Understanding the immune response upon infection with the filarial nematode *Onchocerca volvulus* and the mechanisms that evolved in this parasite to evade immune mediated elimination is essential to expand the toolbox available for diagnostics, therapeutics and vaccines development. Using high-density peptide microarrays we scanned the proteome-wide linear epitope repertoire in Cameroonian onchocerciasis patients and healthy controls from Southern Africa which led to the identification of 249 immunodominant antigenic peptides. Motif analysis learned that 3 immunodominant motifs, encompassing 3 linear epitopes, are present in 70, 43, and 31 of these peptides, respectively and appear to be scattered over the entire proteome in seemingly non-related proteins. These linear epitopes are shown to have an atypical isotype profile dominated by IgG1, IgG3, IgE and IgM, in contrast to the commonly observed IgG4 response in chronic active helminth infections. The identification of these linear epitope motifs may lead to novel diagnostic development but further evaluation of cross-reactivity against common co-infecting human nematode infections will be needed.

## Author summary

Infection with the filarial parasite *Onchocerca volvulus* is the cause of river blindness. We analyzed the immune response against this parasite in infected individuals in order to identify linear epitopes. Using high-density peptide microarrays we discovered three immunodominant motifs in the *Onchocerca volvulus* proteome that induce a broad IgG response, but the typical IgG4 immune response against parasites was absent. Our study led to the identification of novel potential epitope sequences that can potentially be used for detection of infection with *Onchocerca volvulus*.

## Introduction

On the World Health Organization (WHO) list of Neglected Tropical Diseases (NTD), 17 infectious diseases are listed [1], eight of them caused by a helminth infection [2]. Onchocerciasis (or river blindness) is caused by infection with the filarial nematode *Onchocerca volvulus*. More than 99% of infected people live in 31 African countries, while at least 120 million people living in these endemic areas are at risk [3,4]. Diagnostic tools for detection of *Onchocerca* infection traditionally were limited to detection of microfilariae (mf) in small, superficial skin biopsy samples (skin snips) [5]. More recently, a lateral flow assay for the detection of IgG4 antibodies to the parasitic antigen Ov-16 was developed and significantly improved the ease of detection of *Onchocerca* infection [6–9]. However, not all individuals with patent infections are developing (IgG4) antibodies to the Ov-16 antigen [10]. The reason for this is not understood, but it illustrates that a one-single antibody test for epidemiological surveillance purposes is not fully adequate to establish true infection prevalence.

An interesting feature of *Onchocerca*, and more generally, helminth infection is the fact that these organisms are master regulators of the host immune response [11]. In order to maintain a long-term persistence of the parasite in its host, which can last up to 15 years for *O*. *volvulus*, the parasite has developed various strategies to modulate and evade the immune system [12,13]. One important immune evasion strategy is the induction of a highly directed host response known as a “modified Th2-type response”, characterized by the induction of IgG4 accompanied by a decrease in IgE [14–16]. Regulatory T cells (Treg) expressing the transcription factor Foxp3 are involved in modulating the immune response against helminths by reducing the immunopathology via suppression of both Th1 and Th2 responses [17,18].

The availability of complete pathogen genomes allows the identification of new antigenic determinants by using microarray technology, complemented by confirmation in rapid and inexpensive high-throughput serological screening [19]. Current technology allows complete proteome scanning not only for viruses and small bacteria, but now also for larger bacteria or multicellular eukaryotic pathogens, despite their larger proteomes. In the past, identification of linear epitopes in multicellular pathogens’ proteome was limited to a number of candidate proteins after selection by computational methods and *in vitro* evaluation using peptide microarray [20,21]. Here, we describe the results of a screening and confirmation experiments of high-density peptide microarrays containing the entire *O*. *volvulus* proteome with serum samples of *Onchocerca* microfilaridermic patients.

## Results

### Discovery of *Onchocerca volvulus* linear epitopes

**Fig 1:**
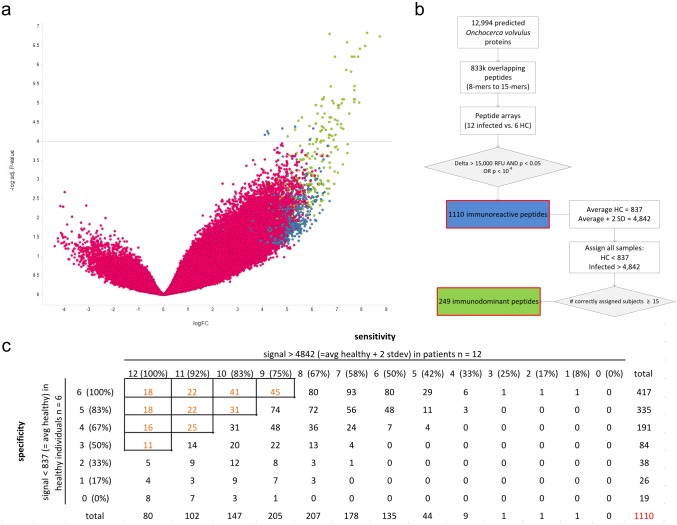
Peptide array analysis of serum samples from *O*. *volvulus* infected individuals. **(A)** Volcano plot showing the log2 fold change in the x-axis and the–log10 adj. p-value in the y-axis for every single peptide analyzed in the arrays. Peptides indicated in blue represent the set of 1110 immunoreactive peptides; peptides indicated in green represent the set of 249 immunodominant peptides. **(B)** Overview of peptide array design and strategy for selection of candidate immunodominant peptides. **(C)** Summary table of the performance of the 1110 immunoreactive peptides. The response for every peptide for the different samples was compared to the preset thresholds (signal >4842 scored as confirmed positive for patients, signal < 837 scored as confirmed negative for healthy controls). These thresholds were based on the signals of the healthy control sample set on the 1110 immunoreactive peptides (as described in Fig 1B).

We designed a high-density peptide chip containing 832,709 peptides, based on the predicted *Onchocerca volvulus* proteome. These peptides were designed as a tiling collection of each predicted protein with an offset of 6 amino acid residues. Most peptides are 15-mers but also shorter peptides were included, with 8mers as the shortest (S1a Fig). Whereas most peptides were uniquely representing one predicted protein, 11,911 peptide sequences were included multiple times, derived from different proteins or protein locations (S1b Fig). These peptides might be part of repeat sequences and/or multiple homologous proteins. Serum samples were selected from 12 Cameroonian Onchocerciasis patients and 6 healthy individuals from Southern Africa (Table 1). Onchocerciasis patients were selected who had at least 2 palpable nodules and 25 microfilaria/mg skin (microfilaridermia). For both sample sets, IgGs were isolated and IgG levels determined (S1c Fig). After standardization to 0.1 mg/mL, seroreactivity profiles were analyzed on the peptide arrays (S1d Fig). The raw data were further processed using rapmad normalization to correct for array- or subarray specific variances. The resulting data set was explored with Limma (Linear Models for Microarray Data) with Benjamini-Hochberg correction in order to determine adjusted p-values and a volcano plot was generated (Fig 1A).

**Table 1:** Characteristics of *O*. *volvulus* infected patients used in peptide arrays.

Subject ID	Origin	Location	Age	Sex	mf/mg skin	# nodules	Onchocerciasis IgG4 LFA
MC197	Cameroon	MBANGA	47	M	100	7	+
MC202	Cameroon	MBANGA	52	M	89	9	+
MC211	Cameroon	MBANGA	54	F	36	2	+
MC234	Cameroon	MBANGA	50	F	70	2	+
MC253	Cameroon	YAMBASSA	54	M	40	6	+
MC260	Cameroon	YAMBASSA	39	F	26	2	+
MC326	Cameroon	DONGO	46	M	45	2	+
MC328	Cameroon	NGOG NGWAS	37	M	74	6	+
MC333	Cameroon	NGOG NGWAS	43	M	300	6	+
MC335	Cameroon	NGOG NGWAS	23	M	30	4	+
MC341	Cameroon	BAMO	22	M	52	5	+
MC343	Cameroon	BAMO	35	M	200	2	-
Ab-E11955	Southern Africa*	unknown	17	F	n.d.	0	-
Ab-E11962	Southern Africa*	unknown	17	M	n.d.	0	-
Ab-E11968	Southern Africa*	unknown	17	F	n.d.	0	-
Ab-E11970	Southern Africa*	unknown	17	F	n.d.	0	-
Ab-E12152	Southern Africa*	unknown	25	M	n.d.	0	-
Ab-E12153	Southern Africa*	unknown	33	M	n.d.	0	-

n.d.: not determined

* Non-endemic area for *Onchocerca volvulus*

As a total of 20,902 peptides were identified that showed statistically significant (i.e. p-value < 0.05) difference in seroreactivity between Onchocerciasis patients and healthy controls, a selection strategy was setup to identify those peptides with the highest diagnostic potential (Fig 1B). In order to identify those peptides with strong antigenicity in the Onchocerciasis patients, a delta value was calculated for each peptide by subtracting the average response in healthy controls from the average response in the patient group. Peptides with delta > 15,000 RFU and p-value < 0.05 or peptides with p-value < 10^4^ were selected. The resulting list of 1110 peptides is considered to represent the linear epitope repertoire of *O*. *volvulus* (S1 File). In order to identify the most immunodominant peptides, for each of these peptides the seroreactivity in the 18 individual samples was investigated. Peptides were selected that correctly assigned the group (i.e. patient or healthy subject) of the sample in at least 15 out of 18 individual samples (Fig 1C). The resulting list of 249 peptides is considered to represent the immunodominant peptides of *O*. *volvulus* (S1 File).

### Three immunodominant motifs are highly present in the immunodominant peptide list

**Fig 2:**
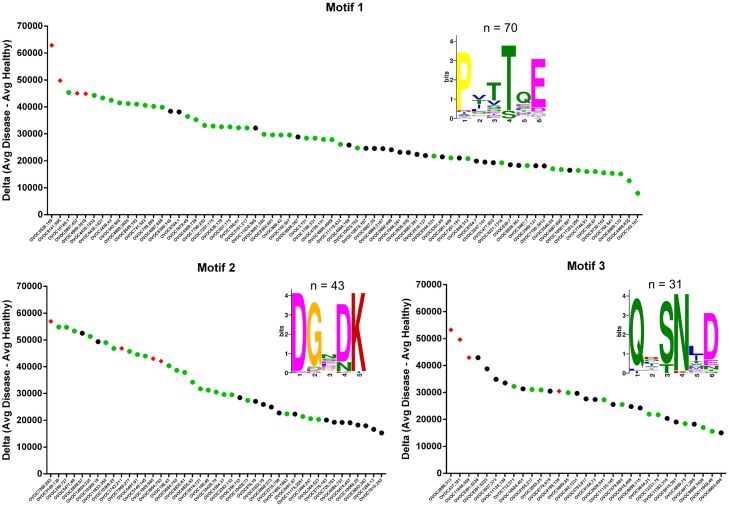
Three immunodominant motifs. For each of the 3 motifs identified, all immunodominant peptides containing one of the motifs (as reported by MEME) are indicated with their corresponding delta value (Avg. Disease–Avg. Healthy Control). Peptides that have been evaluated in peptide ELISA are indicated in green. Peptides that have been further characterized in this study are indicated by red diamonds.

The 249 immunodominant peptides of *O*. *volvulus* were uploaded to the MEME tool in order to determine whether some motifs are overrepresented in these peptide sequences. Analysis of these peptides resulted in the identification of three motifs with very high statistical significance (E-value of 1.2e-067, 2.8e-042, and 3.9e-014, respectively for the 3 motifs) and high occurrence in this list of peptides (n = 74, 39, and 37, respectively for the 3 motifs). Of these peptides, 47, 27, and 15 peptides containing one of the respective motifs have been used in peptide ELISA and all were confirmed to be immunoreactive in (a subset of) *O*. *volvulus* infected individuals (Fig 2). Consensus motifs were determined to be ^1^PxxTQE^6^, ^1^DGxDK^5^ and ^1^Qx(S/T)N(L/I)D^6^ and these consensus motifs were used for further bio-statistical analysis. Using text matching, the number of peptides having a matching for a specific motif was determined in the entire list of 832,709 peptides used in the peptide microarrays. For each of the motifs a confusion table was generated comparing the number of peptides that had significant/non-significant immunoreactivity in the arrays and the presence/absence of the motif in these peptides (Table 2). *Chi-Square* test and *Wilcoxon* test was performed to determine whether the peptides with motifs are enriched in the set of immunoreactive peptides. For all three motifs, peptides with motifs were found to be highly overrepresented in the group of immunoreactive peptides.

**Table 2:** Peptides with motifs are enriched in the set of immunoreactive peptides (confusion table, *Chi-Square* test and *Wilcoxon* test). Between brackets the proportions per row and per column are given.

Motif 1. PxxTQE	**Without Motif**	**With Motif**
**Not immunoreactive**	811805 (100%, 97.49%)	2 (0%, 6.9%)
Immunoreactive*	20875 (99.87%, 2.51%)	27 (**0.13%, 93.1%**)
*χ-square* p-value: 1.49e-205		
*Wilcoxon* p-value: 2.227e-20		
Motif 2. DGxDK	**Without Motif**	**With Motif**
**Not immunoreactive**	811805 (100%, 97.49%)	2 (0%, 4.44%)
Immunoreactive*	20859 (99.79%, 2.51%)	43 (**0.21%, 95.56%**)
*χ-square* p-value: 0		
*Wilcoxon* p-value: 5.104e-31		
Motif 3. Qx(S|T)N(L|I)D	**Without Motif**	**With Motif**
**Not immunoreactive**	811805 (100%, 97.49%)	2 (0%, 11.11%)
Immunoreactive*	20886 (99.92%, 2.51%)	16 (**0.08%, 88.89%**)
*χ-square* p-value: 8.12e-114		
*Wilcoxon* p-value: 3.061e-13		

* Immunoreactive peptides defined as peptides with adj. *P*-value < 0.05 in peptide microarray.

### Conversion to peptide ELISA and determination of sensitivity and specificity

**Fig 3:**
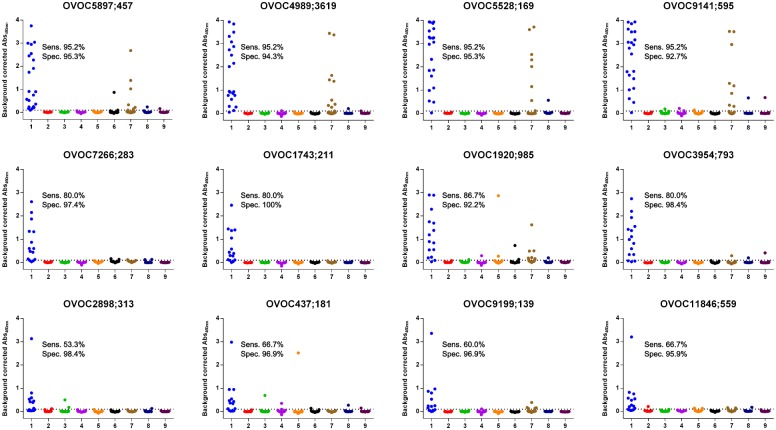
Assessment of immune response against peptides with motifs using peptide ELISA. Immunoreactivity against the 12 selected peptides was determined in a set of 21 *O*. *volvulus* infected individuals (group 1), healthy controls from Southern Africa (group 2), healthy controls from Belgium (group 3) and individuals infected with HIV (group 4), HCV (group 5), Dengue (group 6), *P*. *falciparum* (group 7) and *W*. *bancrofti* (group 8) and asthma patients (group 9).

Peptide microarrays have been described to exhibit higher sensitivity and specificity compared to ELISA [22]. They are however not ideal technology for high-throughput sample testing and definitely cannot be employed at remote areas. We have therefore selected, for each of the three motifs, 4 of the most promising peptides from the microarray data and explored their immunodiagnostic potential by assaying them in an ELISA format (Fig 3). A sample set consisting of 21 (for motif 1) or 15 (for motifs 2 and 3) Onchocerciasis patients was assayed using 200-fold diluted serum in the ELISA. Sensitivity was determined to be 95.2% for all peptides with motif 1, 80.0–86.7% for peptides with motif 2, and 53.3–66.7% for peptides with motif 3 (S2 Fig). In order to determine the specificity of the peptide ELISA’s, sample sets from 10 healthy controls from Southern Africa, 49 healthy controls from Belgium, 25 HIV infected patients, 25 HCV infected patients, 25 Dengue infected patients, 25 Malaria infected patients, 10 LF patients (*Wuchereria bancrofti*) and 25 Asthma patients were assayed. Except for the Malaria infected patients, most samples showed a total absence of signal in the peptide ELISA’s. An obvious response in the Malaria infected patients was however detected for peptides with motif 1. Also OVOC1920;985, a peptide containing motif 2 showed reactivity in 15 of the 193 non-*Onchocerca* infected individuals. Based on all data, specificity was determined to be 92.7–95.3% for peptides with motif 1, 92.2–100.0% for peptides with motif 2, and 95.9–98.4% for peptides with motif 3 (S2 Fig).

### The motifs make up the epitope of the motif-containing peptides

**Fig 4:**
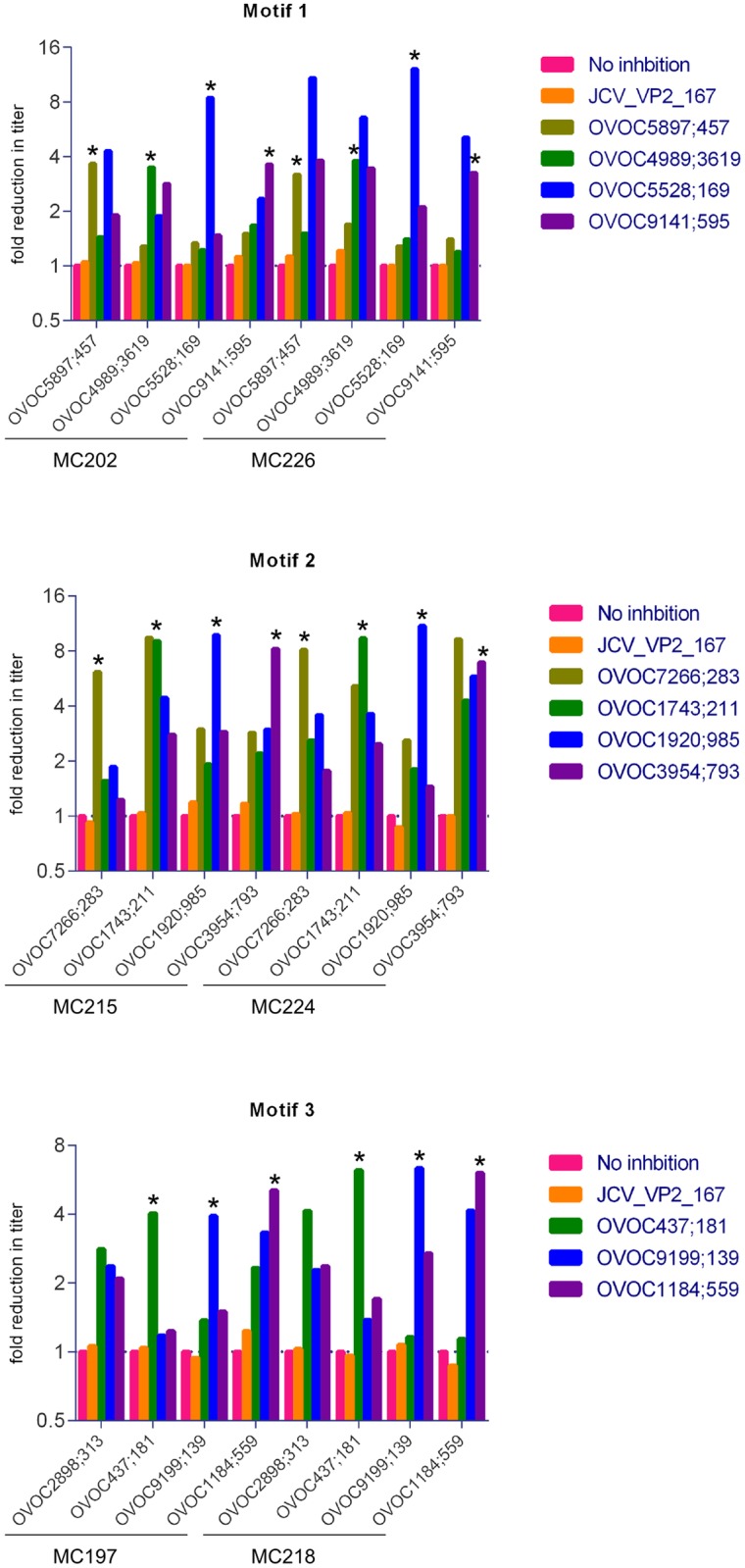
Reactivity in peptide ELISA can be inhibited by peptides with the same motif. Reduction in titer was determined by addition of non-biotinylated peptide to 2 different samples during incubation on the ELISA plates. Plates are coated with the biotinylated peptide as indicated in the x-axis. Titration curves were determined (S3 Fig) and titer was calculated as the dilution showing an Abs_405nm_ that corresponds to 10-fold reduction in absorbance of the non-inhibited sample at the start dilution (i.e. 200-fold). As a negative control, the non-relevant peptide JCV_VP2_167-15mer was included. All self-inhibition results are indicated with *.

Inhibition ELISA assay was carried out to investigate if the peptides with the same motifs bind the same antibodies in Onchocerciasis patients. Two serum samples with high immunoreactivity against the three motifs were assayed in the different peptide ELISA’s in the absence and presence of other *O*. *volvulus* peptides containing the same motif and an irrelevant peptide from JC Polyomavirus (Fig 4 and S3 Fig). All peptides were found to inhibit reactivity against peptides with the same motif, while the irrelevant peptide could not. The specific inhibition effects observed here suggest that peptides with the same motifs share the same epitope, presumably located at the motif site.

**Fig 5:**
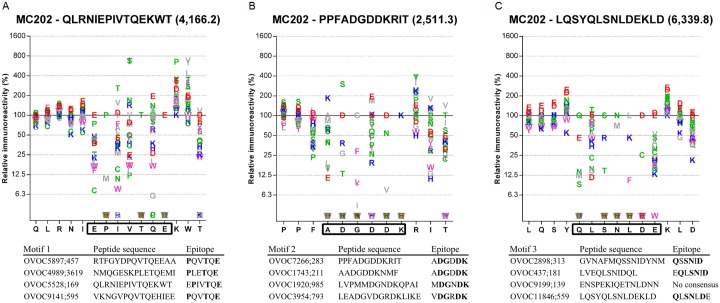
Epitope mapping of peptides with one of three motifs. Amino acid plots using sample MC202 for peptides OVOC5528;169 **(A)**, OVOC7266;283 **(B)**, and OVOC11846;559 **(C)**. Amino acid residues comprising the epitope are indicated by the rectangle on the x-axis. Consensus epitopes determined in 5 samples for the 4 selected peptides for each motif are listed below the plots. Residues indicated in bold represent amino acids essential for antibody binding.

In order to confirm the position of the epitope at the motif site, epitope mapping of the same 12 peptides as described above, was performed. Peptide microarrays covering full substitution scans of all 12 peptides, in which all amino acid positions were substituted by the 19 other amino acids were synthesized and analyzed using 5 different samples from Onchocerciasis patients. For every single peptide and every sample, an amino acid plot was calculated by dividing the spot intensity of a given peptide by the spot intensity of the native peptide (S4 Fig). For each peptide the consensus motif and an example amino acid plot for one sample and one peptide per motif are given in Fig 5. Although some minor differences exist between the different peptides and the different samples, these results clearly indicate that the immunodominant motifs make up the epitope of all tested peptides and in extension, most probably all peptides containing these motifs.

### Isotype profiling demonstrates an IgG1, IgG3, IgE and IgM response

**Fig 6:**
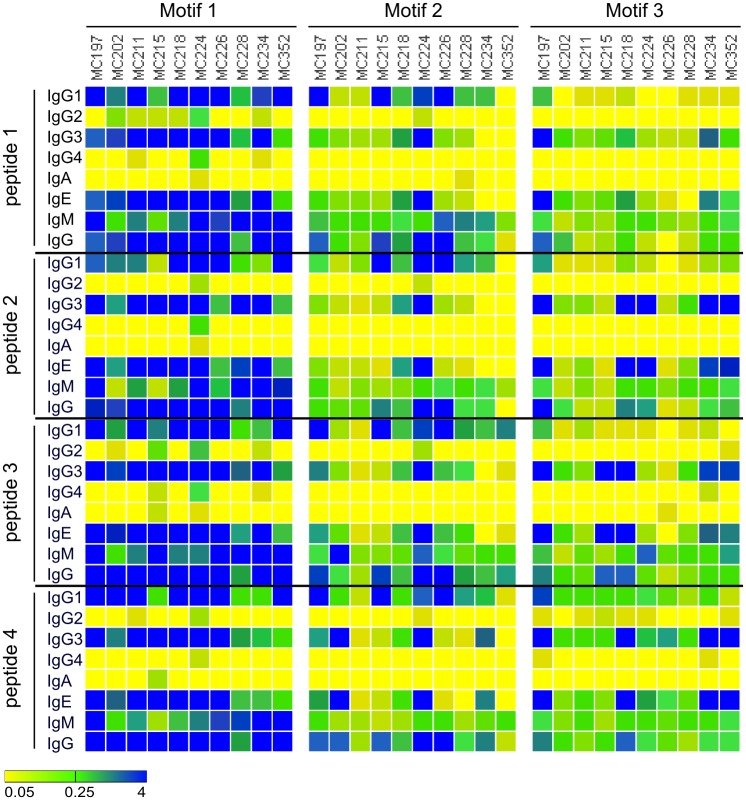
Isotype profiling of the immune response against the peptides containing motifs. Immunoreactivity against the 12 selected peptides was determined in 10 samples using a set of detection antibodies that specifically bind IgG1, IgG2, IgG3, IgG4, IgA, IgE, IgM and IgG. The 4 peptides that were analyzed for motif 1 were OVOC5897;457 (peptide 1), OVOC4989;3619 (peptide 2), OVOC5528;169 (peptide 3) and OVOC9141;595 (peptide 4). The 4 peptides that were analyzed for motif 2 were OVOC7266;283 (peptide 1), OVOC1743;211 (peptide 2), OVOC1920;985 (peptide 3) and OVOC3954;793 (peptide 4). The 4 peptides that were analyzed for motif 3 were OVOC2898;313 (peptide 1), OVOC437;181 (peptide 2), OVOC9199;139 (peptide 3) and OVOC11846;559 (peptide 4). Color scale indicates Abs_450nm_ with yellow corresponding to background absorbance (i.e. 0.05) and blue maximal absorbance (i.e. 4).

Analysis of peptide-specific IgG1, IgG2, IgG3, IgG4, IgA, IgE, and IgM in sera from 10 *O. volvulus* infected individuals was performed with all 12 peptides (Fig 6). Besides IgG1 and IgG3 responses, all individuals had strong IgE and IgM responses against all peptides. In some cases, the IgE response was exceeding the IgG responses. The IgG4 levels in response to the different peptides were low or absent in all tested individuals.

### Occurrence of the immunodominant motifs in other organisms

Using text matching, the proteomes of *Onchocerca volvulus* itself and several other organisms (*Homo sapiens*, *Wolbachia*, *Brugia malayi*, *Wuchereria bancrofti*, *Loa loa*, *Plasmodium falciparum*, *Ascaris lumbricoides*,*Trichuris trichiura*, *Ancylostoma duodenale*, *Necator americanus and Toxocara canis*) were searched for occurrence of the *O*. *volvulus* motifs (Table 3). A detailed list of proteins that were found to contain one of these motifs is presented in S2 File. All three motifs are present several times in all proteomes evaluated. Although these 3 motifs together are present 56 times in *Wuchereria bancrofti*, no reactivity against these motifs was observed in individuals infected with this parasite (Fig 3). For *Plasmodium falciparum* however a clear cross-reactivity was observed with the first motif (^1^PxxTQE^6^). Of interest, this motif has 80 occurrences in the proteome of *Plasmodium falciparum*, with 75 of these occurrences in one protein, the interspersed repeat antigen (FIRA), a protein known to play a dominant role in the human antibody response to *Plasmodium falciparum* [23–25]. As such, this cross-reactivity is not unexpected. The human proteome has a total of 222 occurrences of these motifs but none of them appear to be recognized by the immune system of healthy individuals.

**Table 3:** Number of motifs found in the proteome of *Onchocerca volvulus*, *Homo sapiens*, *Wolbachia*, *Brugia malayi*, *Wuchereria bancrofti*, *Loa loa*, *Plasmodium falciparum*, *Ascaris lumbricoides*,*Trichuris trichiura*, *Ancylostoma duodenale*, *Necator americanus and Toxocara canis*.

	Motif 1 PxxTQE	Motif 2 DGxDK	Motif 3 Qx(S|T)N(L|I)D
***Onchocerca volvulus***	19	29	13
***Homo sapiens***	154	51	17
***Wolbachia***	1	1	0
***Brugia malayi***	12	38	8
***Wuchereria bancrofti***	17	34	5
***Loa loa***	25	42	9
***Plasmodium falciparum***	80	39	7
***Ascaris lumbricoides***	19	33	10
***Trichuris trichiura***	17	33	4
***Ancylostoma duodenale***	36	38	7
***Necator Americanus***	30	27	7
***Toxocara canis***	34	61	13

## Discussion

In this work we describe the identification of the proteome-wide linear epitope repertoire of *Onchocerca volvulus*. Using high-density peptide microarrays, a set of 1110 immunoreactive peptides were identified with 249 of them to be considered immunodominant. Within this set of immunodominant peptides, 3 motifs appeared to be highly overrepresented, suggesting these motifs reflect immunodominant epitopes and are present multiple times in the *O*. *volvulus* proteome. Indeed, both inhibition and epitope mapping studies demonstrated that, upon *O*. *volvulus* infection, substantial part of the immune response is directed against 3 dominant linear epitopes: ^1^PxxTQE^6^, ^1^DGxDK^6^, and ^1^QxSNxD^6^. Detailed examination of the isotypes involved in the immune response against these 3 epitopes demonstrated that this is dominated by IgG1, IgG3, IgE and IgM. This isotype profile is in sharp contrast to the response against other *O*. *volvulus* antigens (e.g. Ov-11, Ov-16, Ov-27, Ov-29, Ov-33) or crude extracts which are characterized by significantly elevated IgG4 antibodies, in some cases even dominating the IgG response [26–30]. Also in the sample set investigated in this study strong IgG4 responses against Ov-16 are observed (both in the Onchocerciasis IgG4 rapid test as in the Ov-16 IgG4 ELISA), which is typically seen in individuals without hyperresponsive phenotype such as in sowda patients [31,32]. The clear IgM responses observed for all 3 epitopes most likely are T-independent and therefore not class-switched, which might be indicative for a constant antigen exposure and constant stimulation of B lymphocytes. Why no IgG4 responses against the 3 dominant linear epitopes were observed is unclear at this moment. Possible explanation might be that these linear epitopes are presented to the immune system as part of proteins or protein fragments that are released by the worm in the bloodstream of the host. This would imply a different antigen presentation in a totally different environment compared to when presented as part of the intact parasite (either adult or microfilaria). As such, the lack of IgG4 responses potentially reflects the difference between conformational (and glycosylated) epitopes on intact surface antigens and linear epitopes exposed to the immune system following their degradation or proteolytic cleavage. This hypothesis is supported by the fact that also for the Ov20 immunodominant antigen only the intact protein can be recognized by IgG4 antibodies, while IgG1, IgE and IgM antibodies were shown to also bind smaller fragments of this antigen [33]. Alternatively, the lack of IgG4 response could reflect the fact that these linear epitopes are not presented to the immune system in an environment that is affected by immune modulatory molecules secreted by the parasite and that are required to switch B lymphocytes towards IgG4 production [15,34–37].

An important point of attention in this work and, in fact, in all studies involving serological assessments, is the possibility of cross-reactivity. The samples that were used for discovery of novel linear epitopes in *O*. *volvulus* were collected in the South West, Littoral and Centre region of Cameroon, regions that are not only endemic for *O*. *volvulus* [38,39] but also for *W*. *bancrofti* [40], *L*. *loa* [41,42], soil-transmitted helminths (*A*. *lumbricoides*, *T*. *trichiura* and hookworms) [43,44] and *M*. *perstans* [45]. It can therefore not be excluded that some of the signals observed in the peptide arrays are in fact due to antibodies induced by these other parasites but cross-reacting with some of the *O*. *volvulus* derived peptides. For the newly identified immunodominant motifs, we have investigated this possible artefact *in silico* by mining the proteomes of these parasites for the presence of these motifs, and also *in vitro* by analyzing samples from patients infected with different pathogens.

When investigating the presence of the identified motifs in other organisms, the interspersed repeat antigen (FIRA) in *P*. *falciparum* was of particular interest. This protein is thought to play an important role in immune evasion and contains highly immunogenic tandem repeat sequences [23–25]. Interestingly, the consensus sequence of these repeat elements (PVTTQE) perfectly matches with the first motif described here (^1^PxxTQE^6^). Next to FIRA, also the erythrocyte membrane protein 1 (PfEMP1) is found to contain both motif 1 (^1^PxxTQE^6^) and motif 2 (^1^DGxDK^6^). This protein is a parasite protein that is exported to the surface of the infected erythrocyte and functions as a ligand for P-selectin during the red blood cell stage of the malarial parasite [46]. Remarkably, this protein is known to induce large amounts of IgG’s and to play an important role in immune evasion by means of antigenic variation [47,48].

Analysis of the peptides containing one of the identified motifs using ELISA in a large set of individuals infected with different pathogens, demonstrated high specificity for *O*. *volvulus*. Except for the group of *P*. *falciparum* infected individuals, almost no responses were observed. Even though the 3 motifs together are present 56 times in *Wuchereria bancrofti*, no reactivity against these motifs was observed in lymphatic filariasis patients from *Onchocerca* non-endemic areas (Sri Lanka and Tahiti). This suggests that *W*. *bancrofti* does not present these linear epitopes to the host’s immune system even though the sequences are present multiple times in its proteome. Similarly, even though the 3 motifs are present 222 times in the *Homo sapiens* proteome, no reactivity against these motifs was observed in non-infected healthy individuals, or in patients with non-related infections (HIV, HCV, Dengue).

The perhaps most obvious implication of this work, is the use of the identified peptides in immunodiagnostic applications. Current standard for serological examination of *O*. *volvulus* infection is the rapid-format tests for the detection of IgG4 antibodies to Ov-16 [6–9]. Although offering ease of detection of *Onchocerca* infection, these tests only have a sensitivity of about 80% [6]. In one study it was even shown that a large percentage of those with onchocercal eye disease living in endemic areas have negative Ov-16 results [10]. Also in the sample set used in this study, only 20 out of 24 (i.e. 83.3%) of the tested individuals were positive using this Ov-16 IgG4 test. This finding demonstrates the need for additional serological markers that can complement the Ov-16 test. All 249 immunodominant peptides identified in this study have the potential to be used for this purpose, either alone or in combination. More research including larger cohorts of *O*. *volvulus* infected individuals, but also cohorts from non-*Onchocerca* endemic areas infected with soil-transmitted helminths, *Loa loa*, *Mansonella* spp. or *Schistosoma mansoni* will however be required to investigate the potential of the identified peptides. Upon demonstration of sensitivity and specificity of these peptides in serological assessment of *Onchocerca* infection, development of point-of-care diagnostic tools, such as lateral flow assay, can be initiated. Besides the confirmation of clinical sensitivity and specificity in this setup, this will also require an in-depth assessment of analytical sensitivity, reproducibility, repeatability, batch-to-batch comparison and comparison with peptide ELISA. Peptide-based diagnostics have been shown to be very useful for serological identification of a plethora of infectious diseases, such as HIV, Hepatitis B Virus (HBV), but also *Borrelia burgdorferi* [49–51]. Immunodiagnostic tests based on peptide serology offer several advantages over others that rely on more complex biological materials. The fact that synthetic peptides are not derived from biological material but are in fact chemically defined antigens greatly simplifies assay standardization and validation [52].

## Materials and methods

### Ethics statement

The following material was obtained through the Filariasis Research Reagent Resource Center (FR3), Division of Microbiology and infectious Diseases, NIAID, NIH: a set of 24 *O*. *volvulus* infected human serum samples, from Dr. Nutman, collected in Cameroon. All samples were de-identified before they were provided to FR3 and usage of these samples for research purposes was approved by the Smith College Institutional Review Board (IRB).

A healthy control panel was composed of 10 serum samples from individuals from Southern Africa, collected in FDA regulated donor centers in the USA and was provided by Tissue Solutions Ltd. (Glasgow, Scotland). Written informed consent was obtained from all individuals, and all samples were decoded and de-identified before they were provided for research purposes. A second panel was composed of 49 plasma samples from healthy individuals from Belgium [53–57]. The Ethics Committee [“Commissie voor Medische Ethiek—ZiekenhuisNetwerk Antwerpen (ZNA) and the Ethics committee University Hospital Antwerp] approved the Protocol, and Informed consent, which was signed by all subjects.

For determination of specificity of the peptide ELISA’s, different sets of plasma samples were obtained from Discovery Life Sciences, Inc. (Los Osos, USA), Bioreclamation IVT (Baltimore, USA), FR3 or Mayo Clinic (Jacksonville, USA). Written informed consent was obtained from all individuals, and all samples were decoded and de-identified before they were provided for research purposes.

### Human serum samples

For the set of *O*. *volvulus* infected human serum samples, information on *O*. *volvulus* infection (number of microfilaria/mg skin and number of palpable nodules) was provided by FR3, along with demographic information (Table 4). All infected individuals had at least 2 palpable nodules and 25 mf/mg skin (microfilaridermia) as determined by skin snip. Sera were collected from clotted blood obtained by venipuncture.

**Table 4:** Characteristics of *O*. *volvulus* infected study population.

Subject ID	Origin	Location	Age	Sex	mf/mg skin	# nodules	Onchocerciasis IgG4 LFA	Ov16 IgG4 ELISA^1^	Used for screening
MC197	Cameroon	MBANGA	47	M	100	7	+	3.74	+
MC202	Cameroon	MBANGA	52	M	89	9	+	2.03	+
MC211	Cameroon	MBANGA	54	F	36	2	+	0.15	+
MC234	Cameroon	MBANGA	50	F	70	2	+	3.93	+
MC253	Cameroon	YAMBASSA	54	M	40	6	+	3.65	+
MC260	Cameroon	YAMBASSA	39	F	26	2	+	n.d.	+
MC326	Cameroon	DONGO	46	M	45	2	+	n.d.	+
MC328	Cameroon	NGOG NGWAS	37	M	74	6	+	n.d.	+
MC333	Cameroon	NGOG NGWAS	43	M	300	6	+	n.d.	+
MC335	Cameroon	NGOG NGWAS	23	M	30	4	+	n.d.	+
MC341	Cameroon	BAMO	22	M	52	5	+	n.d.	+
MC343	Cameroon	BAMO	35	M	200	2	-	n.d.	+
MC215	Cameroon	MBANGA	55	F	45	2	-	0.11	-
MC218	Cameroon	MBANGA	55	F	68	2	-	0.03	-
MC224	Cameroon	MBANGA	60	F	26	3	+	3.34	-
MC225	Cameroon	MBANGA	62	F	75	9	-	0.00	-
MC226	Cameroon	MBANGA	75	F	99	7	+	3.27	-
MC228	Cameroon	MBANGA	57	F	58	2	+	0.68	-
MC235	Cameroon	MBANGA	72	M	38	3	+	n.d.	-
MC319	Cameroon	BAMO	60	M	40	2	+	n.d.	-
MC331	Cameroon	NGOG NGWAS	54	M	78	5	+	n.d.	-
MC352	Cameroon	NGOG NGWAS	60	M	200	14	+	1.86	-
MC360	Cameroon	NGOG NGWAS	66	F	98	6	+	3.74	-
MC362	Cameroon	NGOG NGWAS	70	F	26	6	+	n.d.	-

^1^ Blank-corrected Abs_450nm_

n.d.: not determined

For the two healthy control panels, demographic information is provided in Table 5. An overview of the patient demographics of the specificity panels is provided in Table 6.

**Table 5:** Demographic information of healthy control study populations.

Characteristic	Group	
	HC Southern Africa	HC Belgium
No. of patients	10	49
Age, median (Min-Max)	21 (17–47)	40 (23–59)
Gender, n (%)		
Male	7 (70)	22 (45)
Female	3 (30)	27 (55)

**Table 6:** Demographic information of study population used for determination of specificity.

Characteristic	Group					
	HIV	HCV	Malaria	Dengue	Asthma	LF
Origin	USA	USA	Vietnam	Vietnam	USA	Sri Lanka (8) and Tahiti (2)
No. of patients	25	25	25	25	25	10
Age, median (Min-Max)	n.a.	n.a.	22 (18–40)	26 (4–67)	46(17–91)	33 (13–48)
Gender, n (%)						
Male	n.a.	n.a.	24 (96)	10 (40)	11 (44)	6 (60)
Female	n.a.	n.a.	1 (4)	15 (60)	14 (56)	3 (30)
Unknown	n.a.	n.a.	0 (0)	0 (0)	0 (0)	1 (10)
Source^1^	MC	MC	DLS	DLS	BR	FR3

^1^ MC: Mayo Clinic; DLS: Discovery Life Sciences; BR: Bioreclamation; FR3: Filariasis Research Reagent Resource Center

Abbreviations: LF: Lymphatic Filariasis (*Wuchereria bancrofti*); n.a.: not available

### Peptide microarray design and synthesis

A total of 12,994 predicted *O*. *volvulus* protein sequences were used for array design. These data were obtained from the Wellcome Trust Sanger Institute on August 28, 2014 and can be retrieved from ftp://ftp.sanger.ac.uk/pub/project/pathogens/Onchocerca/volvulus/OVOC_v3.protein.fa. A list of 832,709 peptide sequences was generated from these proteins, by tiling all proteins using a 6 residue offset. Where possible 15-mers were generated, but also smaller peptides, with 8-mers as the shortest, were selected. Additionally, control peptides were included: JCV1 (NLVRDDLPAL), JCV2 (LVRDDLPALTSQE), JCV3 (VRDDLPALTSQEI), EBV (FHPVGEADYFEYHQE) and scramble (WTKTPDGNFQLGGTEP). All controls were included 100 times, randomly positioned on each subarray. Peptide microarray slides (Roche Nimblegen) were divided in 3 subarrays, each presenting the entire set of peptides. One subarray per sample was subsequently used for screening. Peptides were synthesized in situ from C- to N-terminus and Serine was added N-terminally for improved solubility to conduct serum Ig profiling (Nxt-Dx, Vienna, Austria). All peptides were synthesized in 160 cycles. A list of all peptides, including their sequence and position on the arrays is presented in S3 File.

### Human IgG microarray analysis

We used high-density peptide microarrays to screen 12 serum samples from Onchocerciasis patients and 6 serum samples from control individuals from Southern Africa. First, IgGs were purified from 10μL of serum using Melon Gel IgG spin purification kit (Thermo Scientific), following the manufacturers protocol. IgG concentration was determined using the Bradford Protein Assay (Bio-Rad Laboratories), by comparison against a standard curve prepared of purified bovine gamma globulin (Bio-Rad Laboratories). Samples were distributed randomly over 6 slides, containing 3 subarrays. In brief, microarrays were washed once in TBST (Tris-Buffered Saline, 0.05% Tween 20), four times in TBS (Tris-Buffered Saline) and once in H_2_O. Microarrays were incubated at 4°C overnight with 5 μL of purified IgGs (final concentration 0.1 mg/mL) in 1% Alkali-soluble Casein in TBST. The microarrays were washed three times with TBST, and incubated with secondary antibody (Alexa Fluor 647- AffiniPure Goat Anti-Human IgG, Fcγ Fragment Specific, Jackson 109-605-098) diluted 1:10,000 in 1% Alkali-soluble Casein TBST for 3 hours at room temperature. Finally, microarrays were washed three times in TBST, once in H_2_O and dried. Microarray fluorescent-signals were extracted from microarray images, and data sets were subjected to further statistical analysis in order to identify immune reactive peptides.

### Statistical analysis microarray data

Raw intensity data were log2 transformed and normalized using the rapmad normalization procedure [58]. Raw intensities were corrected for array/subarray effect with a correction factor, derived from the linear model on the control peptides.

In order to identify peptides with differential immunoreactivity between the Onchocerciasis patients and healthy subjects, limma (Linear Models for Microarray Data) with Benjamini-Hochberg correction was used [59]. Peptides with limma adjusted p-value < 0.05 were considered to be significantly different between both groups.

Statistical analyses were performed in R version 3.1.2 (2014-10-31).

### Motif identification in the list of immunodominant peptides

The sequences of 249 immunodominant peptides selected from the peptide microarrays were used for motif identification by MEME [60]. The statistically significant (E-value < 10^−4^) motifs with at least 20 hits present in the list of immunodominant peptides were selected for further analysis. The online version of MEME (Version 4.11.2) was used with parameters set to ‘zoops’ (zero or one per sequence) for distribution of motifs, 5 as minimum and 10 as maximum motif width, and 10 as maximum number of motifs to be reported.

### Epitope mapping

Permutation scans were carried out on 12 selected peptides by PEPperPRINT GmbH (PEPperCHIP Platform Technology, Heidelberg, Germany). In a permutation scan, the effect on binding of replacing each of the amino acid residues by all amino-acids is analyzed, which requires the synthesis of 20*x spot peptides per starting peptide (20 amino-acids at x positions, with x the length of the peptide). Five arrays were prepared, each containing the permutation analysis of the WT peptide and of 19 variants. 3,320 different peptides were printed at least in duplicate and were framed by additional HA (YPYDVPDYAG) and polio (KEVPALTAVETGAT) control peptides (186 spots for each control). Each of the peptide arrays was incubated with 500-fold diluted serum sample (MC 202, MC 234, MC 328, MC 333 and MC 335) and stained with Goat anti-human IgG (Fc) DyLight680 (1:5000) and goat anti-human IgM (μ chain) DyLight800 (1:5000). Arrays were scanned with LI-COR Odyssey Imaging System and fluorescence signals were used to calculate relative intensity compared to the native peptide.

### Total IgG peptide ELISA

Biotinylated synthetic peptides were synthesized by standard procedures and purchased from PEPperPRINT GmbH (Heidelberg, Germany). A list of all peptide sequences used in this study is provided in S1 Table.

For determination of peptide specific serum antibody levels peptide ELISA was developed and set up as follows. Streptavidin Coated High Capacity Plates (Thermo Fisher Scientific, Breda, the Netherlands) were rinsed once with 200 μL PBS + 0.05% Tween-20 (washing buffer). Plates were incubated with continuous shaking for 1 hour at room temperature with 100 μL of the selected biotinylated peptides, which were diluted at 1 μg/mL in PBS. In the “no peptide” control wells and positive control wells, PBS was added instead. The plates were rinsed 3 times with washing buffer, thereby eliminating unbound peptide. Then, the different wells were covered with 100 μL of human serum samples, diluted 200-fold in Superblock Blocking Buffer (Thermo Fisher Scientific, Breda, the Netherlands). In “blank” control wells, Superblock Blocking Buffer was added instead, in positive control wells, 25 ng/mL biotinylated HRP in Superblock Blocking Buffer was added. The plate was incubated at room temperature for 1 hour. After incubation, a new 5-fold rinsing cycle was performed as described above. Then, the secondary antibody solution was added to each well. The solution contained an affinity purified Donkey anti-human IgG (H+L) peroxidase conjugate (Jackson Immuno Research Europe Ltd., Newmarket, UK) diluted 1:10,000 in blocking solution. The reaction mixture was incubated at room temperature for 30 minutes. At the end of the incubation period, the plates were rinsed 5 times with washing buffer and treated with 100 μL 1-Step Ultra TMB-ELISA Substrate Solution (Thermo Fisher Scientific, Breda, the Netherlands). After 10 minutes of incubation the colorimetric reaction was stopped with 100 μL 1N HCl. The plate was then read by the SpectraMax Plus 384 Microplate Reader (Molecular Devices, Sunnyvale, USA) at a wavelength (λ) of 450 nm. For each sample, peptide specific signals were corrected for the “no peptide” control signals in order to obtain the background corrected OD values. In case the peptide specific signal was lower than the background, result was adjusted to “0”. A cut-off of 0.1 for background-corrected absorbance was used to determine whether samples were considered positive or negative.

### Isotype determination of the peptide-specific immune response

Peptide ELISAs were performed as described above, except for the secondary antibody used. The following antibodies were obtained from Abcam (Cambridge, UK) and used after diluting 1:10,000 in blocking solution: Mouse monoclonal 4E3 Anti-human IgG1 hinge heavy chain (HRP), Mouse monoclonal HP6002 Anti-human IgG2 Fc (HRP), Mouse monoclonal HP6050 Anti-human IgG3 hinge heavy chain (HRP), Mouse monoclonal HP6025 Anti-Human IgG4 (HRP), Anti-Human IgA antibody [1H9] (HRP), Mouse monoclonal HP6029 Anti-human IgE Fc (HRP), Mouse monoclonal [SA-DA4] Secondary Antibody to Human IgM—mu chain (HRP). Results of isotype determinations are visualized using Gitools (Version 2.3.1).

### Inhibition of the peptide-specific immune response with other peptides

Peptide ELISAs were performed as described above, except that samples were assayed at different dilutions (1:200, 1:400, 1:800, 1:1600, 1:3200 and 1:6400) in order to determine the titer. For peptide-specific inhibition, non-biotinylated peptide (PEPperPRINT GmbH, Heidelberg, Germany) was added to the sample diluent at a final concentration of 50 μg/mL. As negative control, DMSO was added at the same ratio (i.e. 2.5% DMSO). 4-parameter logistic plots were determined using Graphpad Prism (Version 6.02). Titers were calculated by determining the dilution corresponding to an absorbance that equals 10% of the absorbance measured in the non-inhibited sample at a 1:200 dilution (i.e. a 90% reduction).

### Onchocerciasis IgG4 rapid test

The presence of IgG4 antibodies against the *O*. *volvulus* antigen Ov-16 was determined using the SD BIOLINE Onchocerciasis IgG4 test (Standard Diagnostics, Gyeonggi-do, Republic of Korea), according to manufacturer’s instructions. Briefly, 10 μL of plasma was added to the round sample well on the lateral flow strip, immediately followed by the addition of 4 drops of assay diluent into the square assay diluent well. After 1 hour, tests were scored. Tests were considered positive only when both the test and control line were visible. Also faint lines were considered positive, as recommended by the manufacturer.

### Ov-16 IgG4 ELISA

For determination of Ov-16 specific IgG4 levels in serum, ELISA was developed and set up as follows. Maxisorp Plates (Thermo Fisher Scientific, Breda, the Netherlands) were incubated overnight at 4°C with 100 μL of Recombinant Ov-16 antigen for *Onchocerca volvulus* at 1 μg/ml (CUSABIO, College Park, MD, USA). The plates were rinsed once with 200 μL PBS + 0.05% Tween-20 (washing buffer) and incubated at room temperature for 1 hour with 200 μL of Superblock Blocking Buffer (Thermo Fisher Scientific, Breda, the Netherlands). The plates were rinsed 3 times with washing buffer and the different wells were covered with 100 μL of human serum samples, diluted 200-fold in Superblock Blocking Buffer (Thermo Fisher Scientific, Breda, the Netherlands). In “blank” control wells, Superblock Blocking Buffer was added instead. The plate was incubated at room temperature for 1 hour. After incubation, a new 5-fold rinsing cycle was performed as described above. Then, the secondary antibody solution was added to each well. The solution contained a HRP-conjugated Mouse monoclonal HP6025 Anti-Human IgG4 (Abcam, Cambridge, UK), diluted 1:10,000 in blocking solution. The reaction mixture was incubated at room temperature for 30 minutes. At the end of the incubation period, the plates were rinsed 5 times with washing buffer and treated with 100 μL 1-Step Ultra TMB-ELISA Substrate Solution (Thermo Fisher Scientific, Breda, the Netherlands). After 10 minutes of incubation the colorimetric reaction was stopped with 100 μL 1N HCl. The plate was then read by the SpectraMax Plus 384 Microplate Reader (Molecular Devices, Sunnyvale, USA) at a wavelength (λ) of 450 nm.

### Total IgG determination

The total amount of IgG in each serum and plasma samples was determined using the Human IgG ELISA Kit from Sigma-Aldrich (Diegem, Belgium), according to manufacturer’s instructions. Only samples with total IgG levels > 5 mg/mL were considered to be of acceptable quality. Samples that did not meet this criterion were omitted for further analysis.

### Biostatistical analysis of the motifs in the proteome of *O. volvulus* and other organisms

Text matching was used to query the different motifs in the proteome of different organisms. As input file, for *O*. *volvulus* the list of peptides from the peptide microarray was used. For the other organisms, fasta files were generated from the *UniprotKB* database containing all entries for the respective organisms (*Homo sapiens*, *Brugia malayi*, *Wuchereria bancrofti*, *Plasmodium falciparum*). All evaluations were performed in R version 3.2.5 (2016-04-14).

For determination of the overrepresentation of motifs in the immunoreactive peptides, confusion tables were prepared and *Chi square* test and *Wilcoxon* test were performed. Statistical analyses were performed in R version 3.2.5 (2016-04-14).

## Supporting information

S1 Fig Characteristics of the peptides included in the peptide arrays. **(A)** Histogram describing the number of peptides included in the array with different peptide length. **(B)** Number of peptides included in the array with n-fold replication in the array. Remark: the 6 peptides that are included 100 times correspond to the control peptides and are not derived from the *O*. *volvulus* proteome. **(C)** Box plot of the IgG concentration levels in the purified IgG’s for both groups (Disease = *O*. *volvulus* infected). **(D)** Box plots of all signals in the peptide arrays per individual sample. (D = Disease, H = Healthy). Median and 5–95% percentiles are shown.
(PNG)

S2 Fig Sensitivity and specificity calculations of 12 selected peptides, determined using peptide ELISA. (PNG)

S3 Fig Inhibition experiments. (PNG)

S4 Fig Epitope mapping details. (DOCX)

S1 Table Demographic information of the different populations included in this study. (DOCX)

S2 Table List of peptides analyzed in peptide ELISA. (DOCX)

S1 File List of immunoreactive peptides selected from the peptide arrays with their corresponding Delta value, Log FC and adjusted p-value. (XLSX)

S2 File List of proteins from *O*. *volvulus*, *Brugia malayi*, *Homo sapiens*, *Wuchereria bancrofti Plasmodium falciparum*, *Acylostoma duodenale*, *Ascaris lumbricoides*, *Necator americanus*, *Toxocara canis* and *Trichuris trichiura* containing one of the *O*. *volvulus* motifs. (XLSX)

S3 File List of 832,709 peptides analyzed in the peptide arrays with corresponding positions on the array and statistical data (logFC, -log adj. p-value, B and Delta). (ZIP)
