# Supplementary figures and images for: Identification of three immunodominant motifs with atypical isotype profile scattered over the Onchocerca volvulus proteome
Source: PLoS Negl Trop Dis. 2017 Jan 26;11(1):e0005330. doi: 10.1371/journal.pntd.0005330 (PMC5295699; doi:10.1371/journal.pntd.0005330)

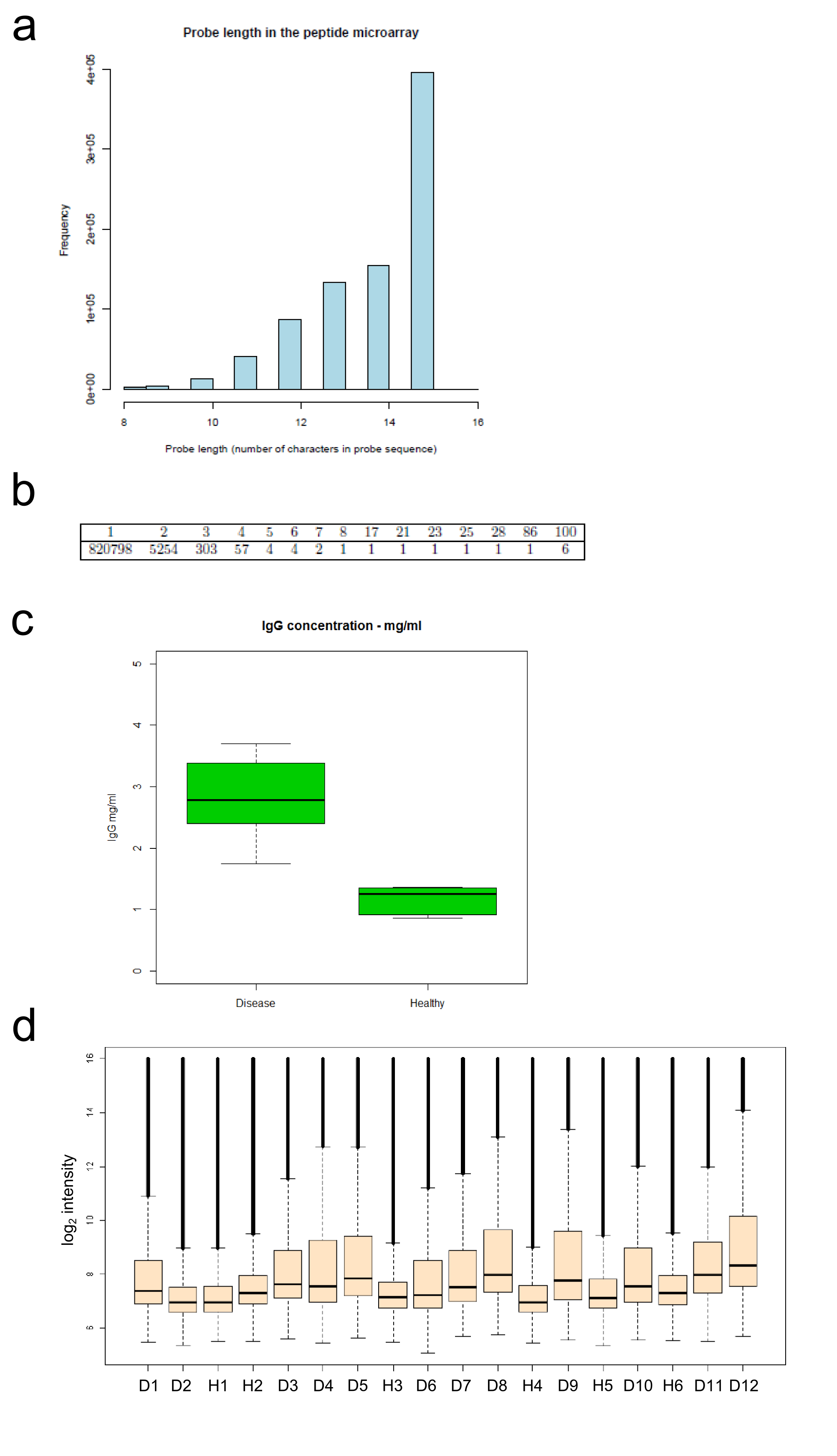

Supplement: S1 Fig — (A) Histogram describing the number of peptides included in the array with different peptide length. (B) Number of peptides included in the array with n-fold replication in the array. Remark: the 6 peptides that are included 100 times correspond to the control peptides and are not derived from the O. volvulus proteome. (C) Box plot of the IgG concentration levels in the purified IgG’s for both groups (Disease = O. volvulus infected). (D) Box plots of all signals in the peptide arrays per individual sample. (D = Disease, H = Healthy). Median and 5–95% percentiles are shown. (PNG) [file pntd.0005330.s001.png]

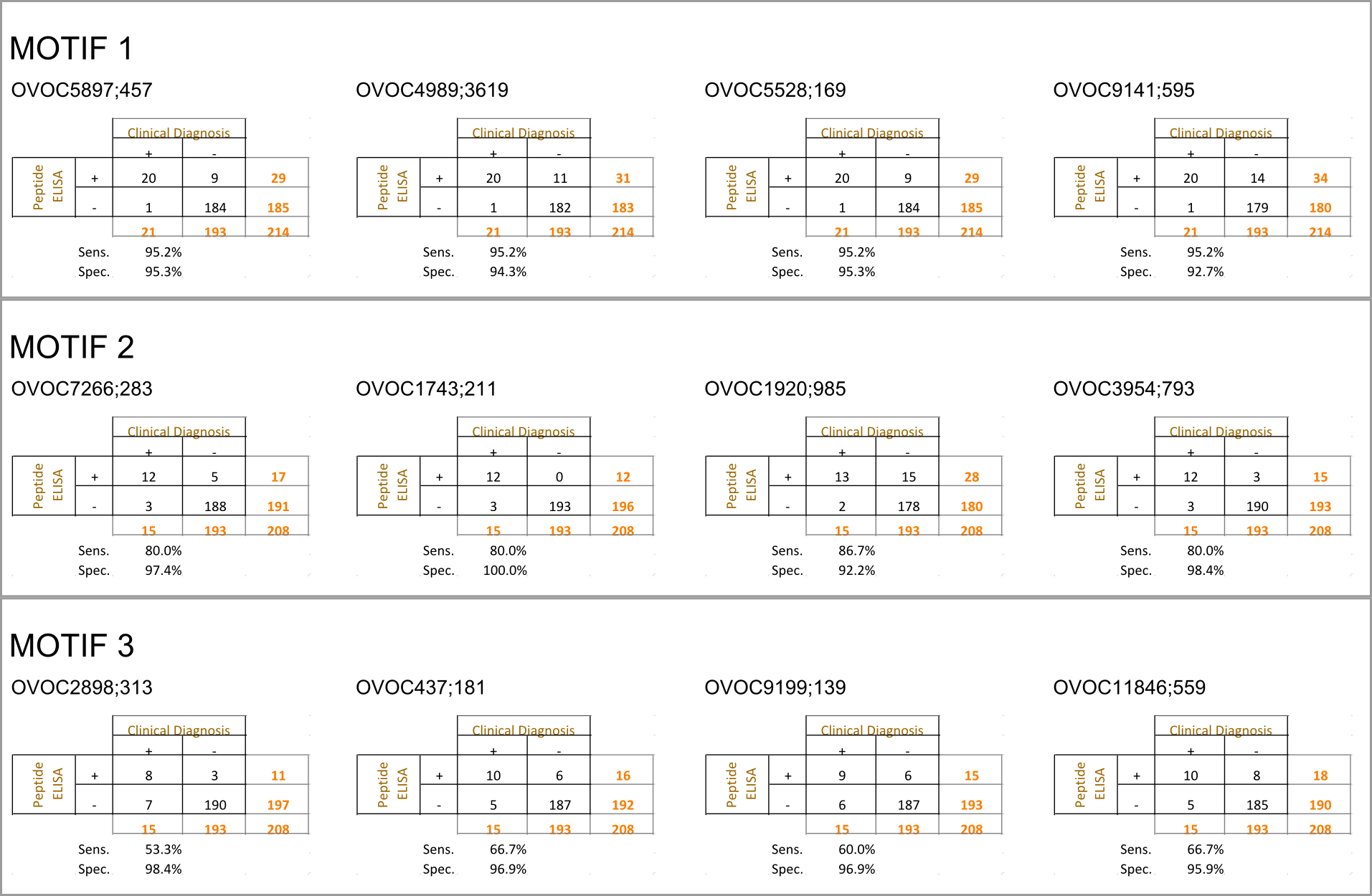

Supplement: S2 Fig — (PNG) [file pntd.0005330.s002.png]

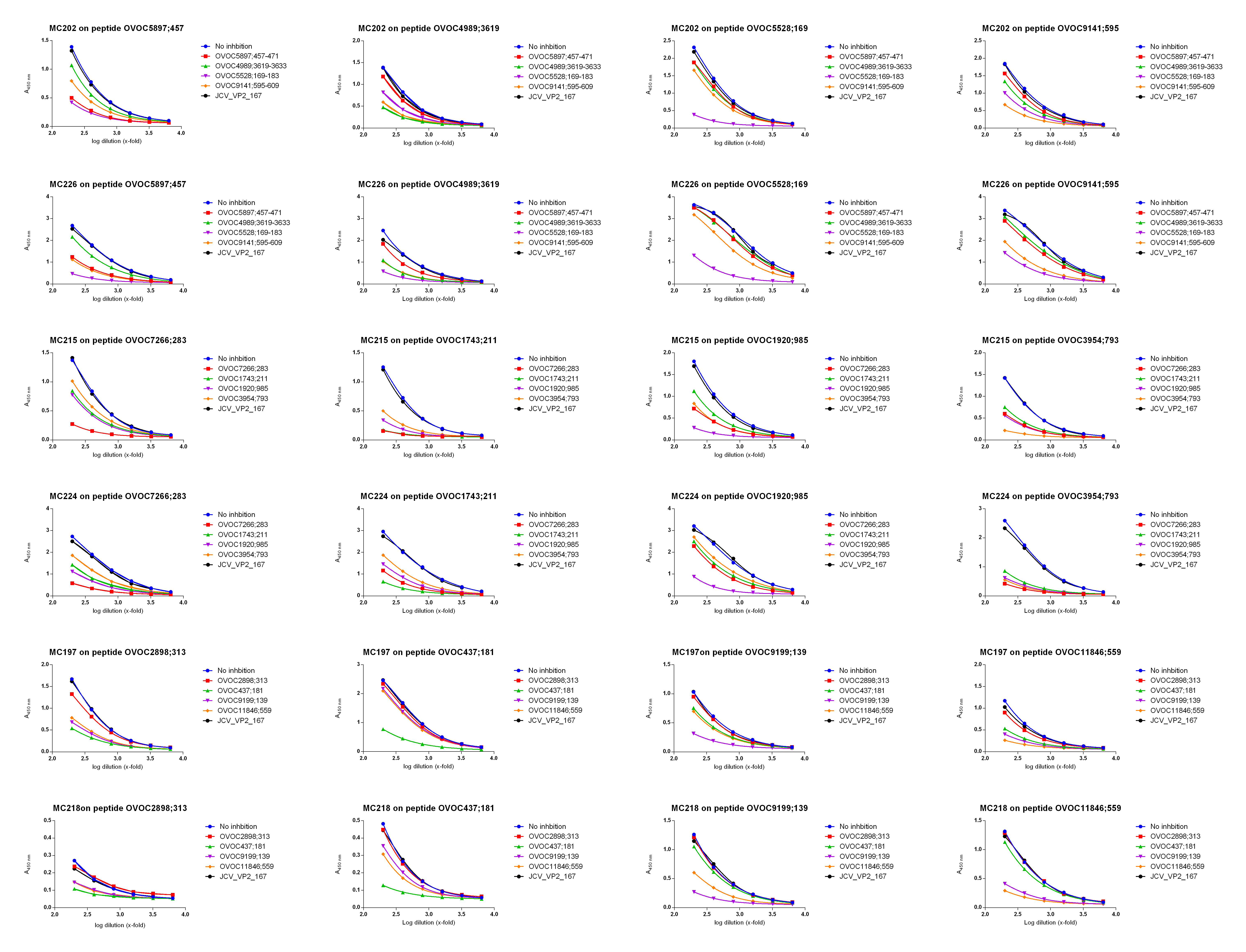

Supplement: S3 Fig — (PNG) [file pntd.0005330.s003.png]

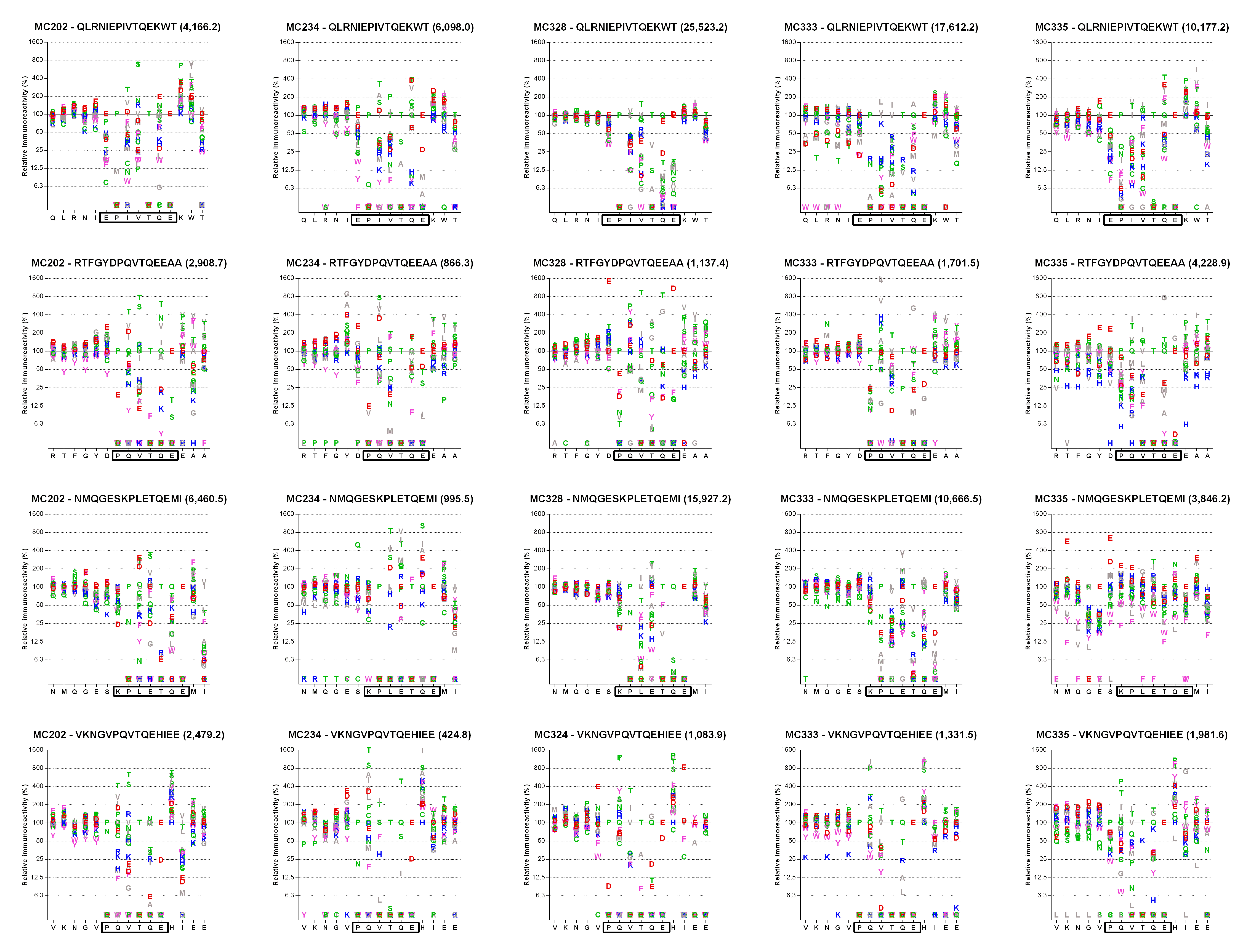


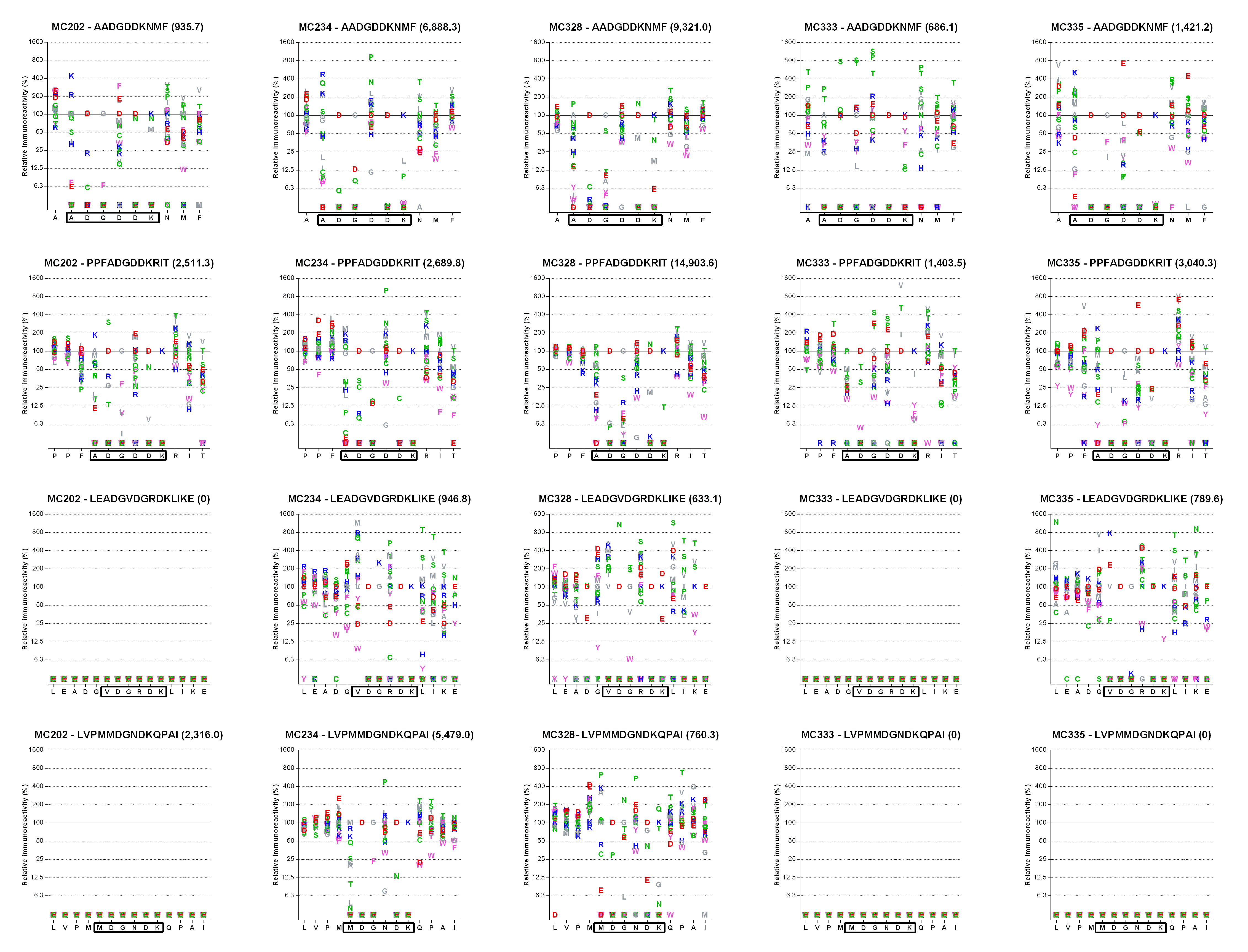


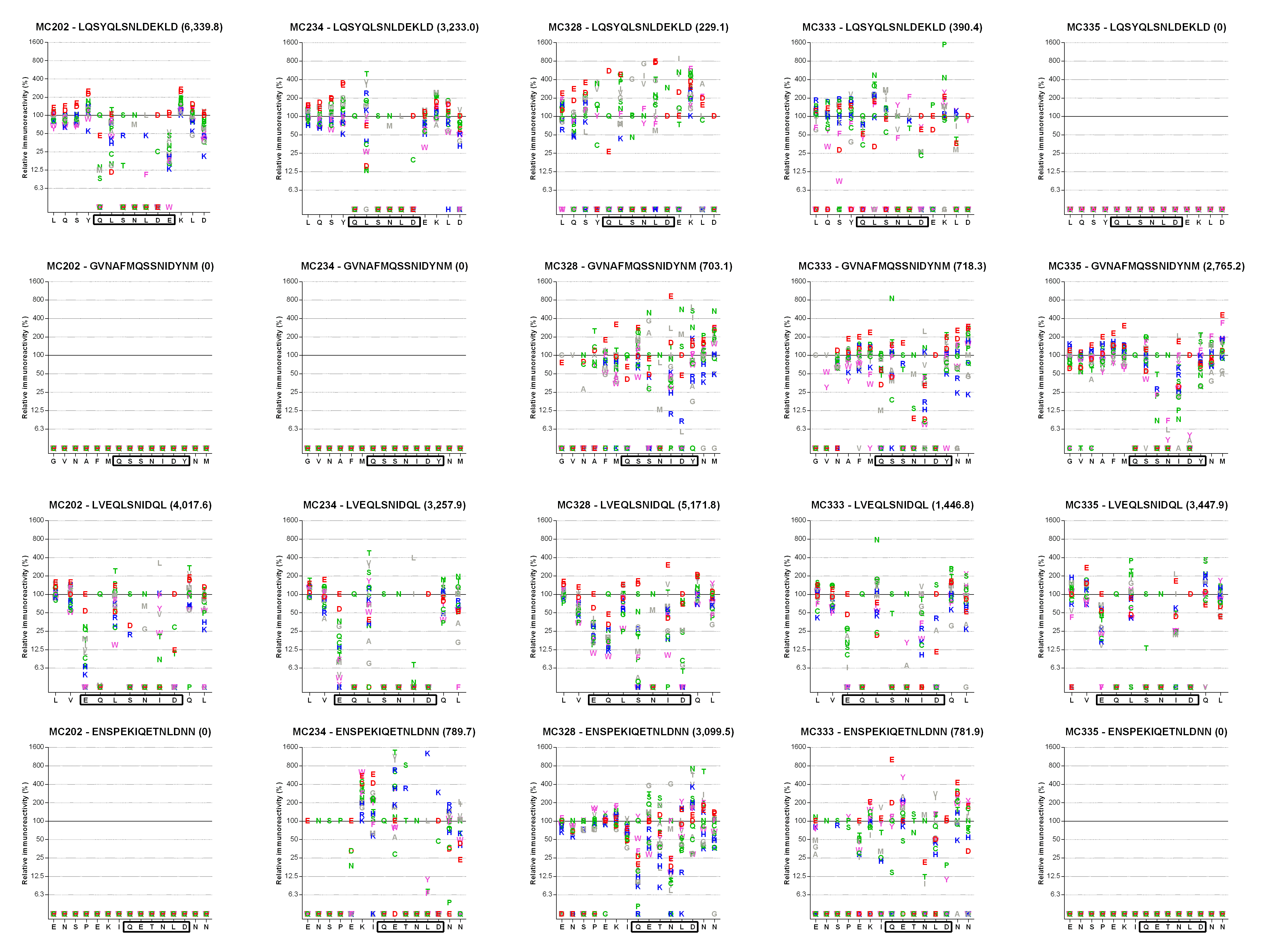

Supplement: S4 Fig — (DOCX) [file pntd.0005330.s004.docx]
