# Supplementary material for: Identification of three immunodominant motifs with atypical isotype profile scattered over the Onchocerca volvulus proteome
Source: PLoS Negl Trop Dis. 2017 Jan 26;11(1):e0005330. doi: 10.1371/journal.pntd.0005330 (PMC5295699; doi:10.1371/journal.pntd.0005330)
Supplement: S1 Table — (DOCX) [file pntd.0005330.s005.docx]

**Supplementary Table S1.** List of peptides analyzed in peptide ELISA.

| **ID** | **Sequence** |
| --- | --- |
| OVOC5897;457 | RTFGYDPQVTQEEAA |
| OVOC4989;3619 | NMQGESKPLETQEMI |
| OVOC5528;169 | QLRNIEPIVTQEKWT |
| OVOC9141;595 | VKNGVPQVTQEHIEE |
| OVOC7266;283 | PPFADGDDKRIT |
| OVOC1743;211 | AADGDDKNMF |
| OVOC1920;985 | LVPMMDGNDKQPAI |
| OVOC3954;793 | LEADGVDGRDKLIKE |
| OVOC2898;313 | GVNAFMQSSNIDYNM |
| OVOC437;181 | LVEQLSNIDQL |
| OVOC9199;139 | ENSPEKIQETNLDNN |
| OVOC11846;559 | LQSYQLSNLDEKLD |
